# What can organisational theory offer knowledge translation in healthcare? A thematic and lexical analysis

**DOI:** 10.1186/s12913-018-3121-y

**Published:** 2018-05-10

**Authors:** Ann Dadich, Navin Doloswala

**Affiliations:** 1 School of Business, Western Sydney University, Locked Bag 1797, Penrith, NSW 2751 Australia; 2 School of Humanities and Communication Arts, Western Sydney University, Locked Bag 1797, Penrith, NSW 2751 Australia

**Keywords:** Knowledge translation, Agency theory, Institutional theory, Situated change theory, Organisational theory

## Abstract

**Background:**

Despite the relative abundance of frameworks and models to guide implementation science, the explicit use of theory is limited. Bringing together two seemingly disparate fields of research, this article asks, what can organisational theory offer implementation science? This is examined by applying a theoretical lens that incorporates agency, institutional, and situated change theories to understand the implementation of healthcare knowledge into practice.

**Methods:**

Interviews were conducted with 20 general practitioners (GPs) before and after using a resource to facilitate evidence-based sexual healthcare. Research material was analysed using two approaches – researcher-driven thematic coding and lexical analysis, which was relatively less researcher-driven.

**Results:**

The theoretical lens elucidated the complex pathways of knowledge translation. More specifically, agency theory revealed tensions between the GP as agent and their organisations and patients as principals. Institutional theory highlighted the importance of GP-embeddedness within their chosen specialty of general practice; their medical profession; and the practice in which they worked. Situated change theory exposed the role of localised adaptations over time – a metamorphosis.

**Conclusions:**

This study has theoretical, methodological, and practical implications. Theoretically, it is the first to examine knowledge translation using a lens premised on agency, institutional, and situated change theories. Methodologically, the study highlights the complementary value of researcher-driven and researcher-guided analysis of qualitative research material. Practically, this study signposts opportunities to facilitate knowledge translation – more specifically, it suggests that efforts to shape clinician practices should accommodate the interrelated influence of the agent and the institution, and recognise that change can be ever so subtle.

**Electronic supplementary material:**

The online version of this article (10.1186/s12913-018-3121-y) contains supplementary material, which is available to authorized users.

## Background

The translation of knowledge from the research-bench to the patient-bedside is pivotal to public health [1, 2]. Yet despite the increasing number of evidence-based practices and the significant allocation of public resources towards them, clinicians do not consistently translate the evidence available to them into patient care [3]. The chasm between the bench and the bedside is partly due to limited use of organisational theory. Despite, greater recognition of the potential value of theory [4, 5], there has been greater attention on ‘how-to’ frameworks and models. Furthermore, the use of theory to explain (expected) observations [6] largely draws from psychology and sociology. Although the utility of organisational theories has been demonstrated retrospectively, their proactive use to inform research design or to analyse data is limited. For instance, following their retrospective application of four organisational theories to published descriptions of the implementation of a training model, Birken and colleagues concluded that organisational theories can offer helpful explanations that are ‘largely untapped’ ([7], p. 12). Consequently, the value of these and other organisational theories – including agency [8] and situated change theories [9] – largely remains unknown. This article helps to redress this imbalance.

This article asks, what can agency, institutional, and situated change theories offer knowledge translation research? This is considered by examining clinician practices when translating evidence-based practices into patient care. Although there are myriad organisational theories that are worthy of scholarship in the context of knowledge translation [10, 11], these three were purposefully selected because – according to Estabrooks and colleagues ([12], p. 33) – they collectively represent ‘adjuvant’ and ‘necessary’ theories to better understand and ultimately improve knowledge translation. This is because they collectively offer, ‘an armamentarium of maps to navigate the knowledge-translation field’, detail for which are provided in the explication of the theoretical lens. As such, using two complementary approaches to analyse clinician interviews to optimise rigour – namely, thematic and lexical analyses – the study demonstrates how this tri-focal theoretical lens revealed different aspects of this complex process. More specifically, it reveals: (1) tensions between the clinician, their organisations, and their patients (as per agency theory); (2) the role of clinician-embeddedness within their specialty, their profession, and their organisation (as per institutional theory); and (3) the role of localised adaptations over time (as per the situated change theory). This study is both theoretically and methodologically novel. Theoretically, it is the first to bring agency, institutional, and situated change theories together to what has remained an intractable issue in healthcare. Methodologically, this article is the first to use thematic and lexical analyses to examine the research material in different ways to better understand the contribution of each theory, given the overarching research question. While thematic analysis enables ‘a focus on meanings and a better connectivity within the data to show how one concept may influence another’ ([13], p. 231), lexical analysis offers ‘a “helicopter” view of the data’ ([14], p. 2). This novelty has implications for theories of knowledge translation and how this process might be examined.

This article begins with an overview of knowledge translation. It then describes agency, institutional, and situated change theories to examine and understand it. To clarify the potential value of these theories – as per the overarching focus of this article – they were used to examine the implementation of healthcare knowledge into practice. More specifically: reflecting agency theory, the study examined how clinicians negotiate their relationships with their organisation and their patients (to whom they are accountable) to translate knowledge into practice; reflecting institutional theory, the study examined how clinicians adapt to the expectations and demands of their profession and the practice in which they worked; and reflecting the situated change theory, the study considered how clinicians exercise practice change. The article concludes with a discussion of the theoretical contribution of this study and the transferability of lessons garnered.

### Knowledge translation

Despite the myriad terms to refer to knowledge translation, it might be understood as ‘any activity or process that facilitates the transfer of high-quality evidence from research into effective changes in health policy, clinical practice, or products’ ([15], p. 355). Although the ultimate aim is to use (near) irrefutable evidence to improve patient care, this translation (*translation* being the operative word) is influenced by: clinician expertise; patient (and potentially carer) preferences; available resources; and the context of care – this might include a consideration of the team the clinician collaborates with (within and beyond the organisation), leadership style(s), organisational culture, the political climate, and local epidemiology, among others [16].

Given the interrelated and dynamic relationship between the aforesaid factors, knowledge translation can hardly be described as linear [17]. This is suggested by some of the models developed to understand the complexity [18]. Despite their potential value, the models are generally light on theory [19]. While they might help to describe knowledge translation and reveal the factors that help and hinder related processes, they have a limited capacity to explain, predict, or control phenomena. This was indicated by Davies and colleagues [20], who found that only 6 % of studies included in their systematic review used theory to guide the design and/or implementation of interventions to facilitate knowledge translation – notably, cognitive theories, like social cognitive theory, and theories of learning, including social learning theory. This leaves knowledge translation research at risk of theoretical paucity, with limited opportunity to understand and address the chasm between the bench and the bedside. This article addresses this by determining what organisational theory can offer knowledge translation research.

As a seemingly complex and intractable issue, knowledge translation is likely to require a different approach – theoretically and (related to this) methodologically. This supposition follows Greenhalgh and Wieringa who argued for research that: (1) investigates how clinicians ‘balance… [a] generic recommendation… against the particularities of a case in the here-and-now’ ([21], p. 508); (2) examines the ‘development and activity of communities of practice’ to unpack the complex and wealth of knowledge that clinicians draw from – this includes knowledge that is ‘explicit and tacit, general and specific, acquired over a lifetime of learning, reading and experience’; (3) draws from critical management studies to reveal the power dynamics that influence understandings of knowledge, as well as its construction, acceptance, and use; (4) explores ways to facilitate interaction between multiple forms and sources of knowledge; and (5) considers ‘the cycle of developing, implementing and revising clinical guidelines in a way that recognises and captures practical wisdom and case knowledge’. To address these calls in part, this study uses a theoretical lens that draws from the ‘adjuvant’ and ‘necessary’ theories of agency, institutional, and situated change theories ([12], p. 31).

Guided by these theories, this article draws on their explanatory power to clarify and explain how general practitioners (GPs) translate clinical knowledge into practice. Using sexual healthcare as the focus of the study, this was achieved through a longitudinal study that involved two analytical methods to: (1) identify sources of information on evidence-based sexual healthcare among GPs; and (2) examine how this knowledge was translated into practice. To optimise rigour, the study involved: the collection of qualitative and quantitative data; three analysts (each with a distinct perspective); and two analytical methods – namely, researcher-driven coding and lexical analysis, which is relatively less researcher-driven. The study was also longitudinal as it involved data collection at two time-points. Before presenting this research, the following section provides a brief overview of the tri-focal lens used to examine knowledge translation processes and a rationale for its use.

### Theoretical Lens

The theoretical lens adopted in this research is comprised of the agency, institutional, and situated change theories. Each is described and critiqued as follows (see Table 1 for a summary).

**Table 1:** Comparison of Agency, Institutional, and Situated Change Theories^a^

	Agency Theory	Institutional Theory	Situated Change Theory
Key idea	Organisational practices arise from efficient organisation of information and risk-bearing	Organisational practices arise from imitative forces and firm traditions	Change occurs through frequent, emergent, and sometimes imperceptible variation
Basis of organisation	Efficiency	Legitimacy	Subtlety
View of people	Self-interested rationalists	Legitimacy-seeking satisficers	Trialists
Role of environment	Organisational practices should fit environment	A source of practices to which organisation conforms	Organisational practices should negotiate with environmental conditions
Role of technology	Organisational practices should fit technology employed	Technology moderates the impact of institutional factors or can be determined institutionally	Technology is appropriated to organisational conditions
Problem domain	Control problems (vertical integration, compensation, regulation)	Organisational practices, in general	The dynamic interplay between innovation, people, and their organisational context
Independent variables	Outcome uncertainty, span of control, programmability	Industry traditions, legislation, social and political beliefs, founding conditions that comprise the institutional context	Organisational context, individual interests and capacities
Assumptions	People are self-interested, rational, and risk-averse	People satisfice and conform to external norms	People are innovative, and perseverant

^a^ *Adapted from Eisenhardt* [22] *and Orlikowski* [9]

#### Agency theory

Agency theory awards primacy to the politic of the individual or agent, highlighting the role of enticements and personal agendas – in brief, ‘It reminds us that much of organizational life, whether we like it or not, is based on self-interest’ ([8], p. 64). This understanding can help to determine the most appropriate type of agent-principal relationship or contract to optimise performance and manage risk – more specifically, when might a contract be used to efficiently shape behaviour, rather than a contact (partly) based on outcomes [22]? For instance, agency theory has been used to understand how the pharmacist role might be expanded and sustained to one of prescriber [23] and how governing hospital boards engage in quality improvement practices [24]. Agency theory holds particular relevance to research on healthcare organisations because of the strong professional identity within medicine [25].

Professional identity is an awareness of the role and functions that one performs or is expected to perform in a social context as a member of a particular profession. It essentially concerns the self and the self in relation with others – be they individuals or organisations [26]. Professional identity is an ongoing concern of the professional involving work practices, social interactions with colleagues and clients, and sense of place within the professional institution and the professional discourse. Professional identity is thus socially bestowed, socially sustained, and socially transformed. Given such reinforcement, professional identity is typically enduring [27]. Within medicine, professional identity appears particularly robust, as demonstrated by the value placed on professional autonomy and efforts to retain it. Given the strong professional identity within medicine, the unit of analysis within this study is the clinician (as agent), and their perceptions of their obligations (or contract) with those they are accountable – namely, their organisation and their patients (as principals; [8]).

However, agency theory is not without criticism. Its capacity to theorise performance has been questioned [28]. It is faulted for its limited accuracy and generalisability, partly due to a perceived reliance on contract design to reduce uncertainty, with limited recognition of social dimensions. This is indicated by references to the ‘problem of unilateralism’ [29] and ‘boundary conditions that limit the theory’s explanatory power of current business phenomena’ – notably, strategic and social entrepreneurship, family businesses, and modern stakeholder relations ([30], p. 182). However, developments that redirect the theoretic gaze from the agent to the organisation and institution serve to address (some of) these critiques – these include: an argument to recognise the complete structure of agency [31]; a repositioning of the firm as a body of agents who endeavour to harmonise their efforts towards a shared goal [32]; an empirical expansion from narrow to demarcated self-interest [33]; and recognition of a bilateral relationship between agents and principals [29]. This (re)interpretation suggests agency theory, ‘is particularly suited to [help to] bridge the gap between theory and application’ ([31], p. 94). Towards this aim, and guided by a rehabilitated theory [32], this study centres on the human agents.

#### Institutional theory

Institutional theory awards greater primacy to the institution – its rules, requirements, customs, and conventions; that is, its ‘rules of thumb’ ([22], p. 489). These standardisations and assumptions are beneficial for two reasons. First, they foster efficiencies – stock-standard practices enable individuals to rely on experience when attending to mundane or low-priority matters, and direct their energies to novel or high-priority issues [34]. Second (and perhaps more importantly), they offer legitimacy. Rules, requirements, customs, and conventions help organisations to operate, orchestrate, and evaluate [35]. These activities can suggest responsible management [36] and thus shape the beliefs and values of those within the organisation as well as those external to it.

Despite these seeming benefits, an organisation’s ‘rules of thumb’ can also represent a constraint. In the absence of a systemic overhaul, change can be difficult to introduce and sustain [37]. Individuals who are tightly wedded to, or embedded within their social context are largely said to resist change [38]. Although personal agency can help to transform embeddedness into an opportunity for change, albeit incrementally [39], such change still requires negotiation with prevailing organisational norms.

Following the institutional turn in organisational research, reinforcing that institutions are worthy of scholarship [40], there have been calls to refocus institutional theory on its primary focus [41] – namely, to understand how, ‘social structures and processes… acquire meaning and stability in their own right rather than as instrumental tools for the achievement of specialized ends’ ([42], p. 1147). The scholarly scales have tipped towards accounts of typically formalised institutions and the processes therein, much to the neglect of how an organisation operates [43]. Consequently, institutional theory has become diluted, explaining much about legal entities with limited critical insight [44].

To redress this imbalance, there is a need to consider institutional stories [45] – that is, sensemaking processes, particularly those at the individual level, to capture different forms of organisations, and of organising [46]. Recognising a fluid relationship between institutions and individuals [47], this is likely to involve interpretivist methods to examine how organisational actors experience institutions, and how they adopt and justify non-rational practices that seem at odds with economic, isomorphic motivations. As such, this study involved the collection and analysis of qualitative material to elucidate participant accounts of knowledge translation processes and how they interpreted or made sense of these processes.

#### Situated change theory

As its name suggests, the situated change theory suggests that change is situated in an evolving context, in which individuals adapt or metamorphosise accordingly. Given its strong links to the environment, change is gradual and meaningful, rather than planned and deliberate. Change involves subtle deviations from the paths most travelled and most controlled to pursue a flexible and self-organised path [48]. And only by peering over one’s shoulder, do the paths and patterns become apparent [49]. The metamorphosis of practice change reflects the ways in which individuals make sense of their world [9]. Consider for instance, the ways that situated change theory has helped to understand how individuals improvise with, and adapt the technologies they work with [50]. Research reveals technology-use as an emergent activity, shaped by previous practices, the properties of the technology, political and social dynamics, as well as the task at hand [51]. For two reasons, unscheduled, incremental change can be advantageous, particularly when confronted by demands for change. First, the respite between periods of change can help to reduce stress [52] – it offers a breathing space. Second, respite also represents a time to master new skills [53], which is likely to be easier with reduced stress [54].

Given significant change within many national health systems – including an ageing population, the rise of chronic and preventable diseases, technological developments that enable medical breakthroughs, and the rise of healthcare costs [55] – the situated change theory is likely to hold particular relevance to the study reported in this article. In the wake of the aforesaid changes, governments are vying for different ways to improve the organisation, management, and delivery of healthcare, notably via healthcare reforms. Given the rate of reform, it might be argued that reform is now routine [56]. However, reform often demands considerable change. It requires organisations to restructure, downsize, merge, close, or (perhaps more to the point), do more with less. These sizeable demands suggest that change at the individual level might be eased by opportunities for respite – when there is opportunity to reclaim a degree of autonomy and remain unchanged, like that suggested by the situated change theory.

#### Relevance to knowledge translation

**Fig. 1:**
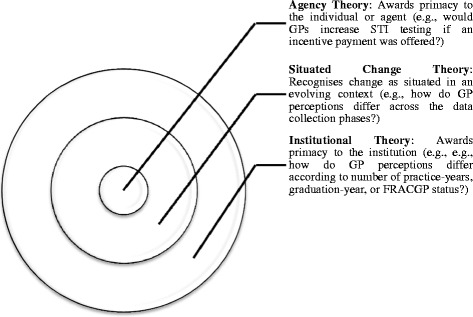
Relationship between Agency, Institutional, and Situated Change Theories

In addition to representing ‘adjuvant’ and ‘necessary’ theories to further knowledge translation research ([12], p. 33), the agency, institutional, and situated change theories each direct the scholarly gaze to interrelated aspects of an organisation. Akin to the layers of an onion (see Fig. 1), institutional theory clarifies (some of) the parameters of practice – the field in which an organisation operates and the forces that shape what it, and those therein, can and cannot do. Despite these parameters, agency theory highlights the influential role of self-interest. Like an inner layer of the metaphorical onion, it helps to moderate the influence of these forces by underscoring personal agency. Yet this is not to suggest causal relationships between institutional or personal factors and behavioural (or attitudinal) change – for situated change theory suggests that change is an evolution, which occurs through dynamic relationships between factors, structures, and processes; furthermore, this evolution only becomes apparent over time.

Despite their complementarity and relevance to knowledge translation research, the use of the agency, institutional, and situated change theories in this study is not intended to suggest the irrelevance or insignificance of other theories (or frameworks) – organisational or otherwise. Indeed, although beyond the scope of this study, the relevance of theories that direct scholarship to the antecedents that trigger innovation, the processes through which innovation is introduced, and/or the outcomes associated with innovation, have been acknowledged – consider for instance, the diffusion of innovation theory [20]. Also recognised is the value of social theories, including (but not limited to) social capital theory and network theory [12, 57, 58]. And like these, the agency, institutional, and situated change theories have received limited scholarly attention in the context of knowledge translation. As such, and heeding calls for the proactive use of organisational theories [7], a study was conducted, guided by this tri-focal lens, to examine how GPs translate clinical knowledge – particularly evidence-based sexual healthcare – into practice. This knowledge was presented to the GPs in the form of a clinical aide – namely, the STI Testing Tool (withheld for blind-review [2, 59, 60]).

## Methods

### Participant recruitment

Following clearance from the relevant university ethics body, GPs affiliated with at least one Division of General Practice in NSW who were practicing within this Australian state were invited to contribute to this study. Divisions were professional bodies that supported GP-members through the provision of training, resources, and opportunities to collaborate with other health professionals [61]. Participants were recruited in three ways. First, three Divisions in Greater Western Sydney informed its members about the study and the opportunity to participate via its website and/or newsletters that were circulated to members. Second, the lead author attended workshops and seminars hosted by these Divisions to personally inform attending members about the study and the opportunity to participate. Third, GPs who participated in this study were invited to inform their GP colleagues about the study and the opportunity to participate [62].

### Research context

The context of this study is sexual health. Sexual health was an appropriate focus for two reasons. First, despite the prevalence of sexually transmissible infections (STIs) and the availability of evidence-based guidelines, the delivery of sexual healthcare is limited, particularly among GPs [63]. The apparent disconnect between primary health *needs* and the delivery of primary *healthcare* indicates that sexual health is an appropriate focus to examine the translation of medical knowledge into practice. Second, the primary care sector in many Western nations is experiencing significant reform, the aim of which is to shift the focus from hospital care to primary care [64]. Given the high-costs associated with a hospital-centric health system, government efforts to redress this imbalance see greater focus (if not pressure) on community-based care – this includes the primary care sector. These two reasons lend sexual healthcare as an appropriate context for this study.

### Data collection

A semi-structured, open-ended interview schedule was designed to explore the factors that help or hinder the use of evidence-based practice in the delivery of sexual healthcare. Reflecting the tri-focal theoretical lens, this included an examination of: personal interests at the micro level, thereby reflecting agency theory (e.g., learning preferences, patient-communication style); institutional forces at the meso (e.g., practice management) and macro levels, thereby reflecting institutional theory (e.g., Division support); as well as the dynamics between these interests and forces that shape attitudes and behaviours over time, thereby reflecting the situational change theory. To understand whether and how the STI Testing Tool was translated into practice, interviews were conducted twice – before the GPs were provided with the STI Testing Tool, and then approximately three to four months later, depending on GP availability (see the Additional file 1 for the interview schedules). As such, data were collected from the same sample of participating GPs on two occasions. The timeframe for data collection was thought to provide adequate time for perceived change. Interviews transpired for approximately thirty to sixty minutes and were digitally recorded; recordings were then transcribed verbatim for analysis. To ease data collection, both interviews were complemented with a closed-item survey. The survey included personal demographic items; organisational demographic items; items regarding professional development activities; clinical vignettes; and items regarding initiatives that would bolster the use of evidence-based practice.

### Data analyses

Guided by the theories reviewed earlier, and using the individual as the unit of analysis, the analysis of the interview transcripts involved two interrelated phases – thematic, which was relatively researcher-driver, and lexical, which was relatively program-driven. These two phases were conducted to view the research material in distinct ways and thus reveal the strengths and shortcomings of the theories within this context. As such, although data collection occurred twice, and while data were sourced on different variables, the analysis focused solely on areas deemed relevant by the tri-focus lens.

#### Thematic analysis

The thematic phase involved three overlapping processes: (1) constant comparison analysis [65]; (2) the optimisation of variance [66]; and (3) triangulation through researcher and member checks [67]. Aided by QSR NVivo, constant comparison analysis involved methodically coding the material and constructing themes from the codes [68]; themes were identified in relation to the study aim and objectives, and were compared between GPs. To protect GP identity, names and identifying characteristics are not disclosed. The optimisation of variance involved ensuring that descriptions and explanations about the research material contained both typical and atypical elements [66] – that is, similar themes (e.g., the types and sources of clinical knowledge) as well as those that were unique (e.g., organisational strategies that promote sexual healthcare). Triangulation involved the participation of three individuals in the analysis [67] – two researchers (one of whom is relatively unfamiliar with relevant research) and one Division representative. Discussion of their constructed themes helped to increase the rigour and trustworthiness – or dependability [69–72] – of the findings. These three interrelated processes helped to ‘decrease, negate, or counterbalance the deficiency of a single strategy, thereby increasing the ability to interpret the findings’ ([73], p. 253).

#### Lexical analysis

The lexical phase involved conceptual and relational content analysis [74]. To optimise the likelihood of a methodical approach [75], this process was aided by Leximancer – data-mining software that uses Bayesian reasoning to detect key concepts and reveal their relationships [76]. Using algorithms, Leximancer identifies frequently occurring and co-occurring words and amalgamates these to form and visually map concepts that reflect themes within the text [77]. The maps convey three types of information – ‘the main concepts in the text and their relative importance; the strengths of links between concepts (how often they co-occur); and similarities in contexts where links occur’ ([78], p. 1735). Clusters of concepts within a map suggest contextual similarity. The components of these concepts are ordered within a thesaurus, comprised of relevant words and weightings to indicate relative importance. By default, two-sentence blocks of text are then assessed to identify evidence of conceptual relevance – however, given the concise nature of some responses, one-sentence blocks of text were assessed. For further detail on Leximancer and its validation, relative to other coding practices, see Smith and Humphreys [79].

Following Angus-Leppan and colleagues [74], the recommended Leximancer analysis procedure was used. This involved aggregating the transcripts from both stages of data collection and using the discovery to determine the concepts automatically generated by Leximancer. This helped to determine how the data might be refined and how relevant concepts were profiled. For instance, it was evident that interviewer comments dominated certain concepts. For this reason, all interviewer quotes were suppressed to give voice to the GPs. Although this approach potentially discounts the interviewer-interviewee exchange and context, this was addressed by sourcing the full transcript when analysing relevant concepts.

Preparing the interview transcripts for analysis involved two steps. First, given the focus of this study on knowledge translation, the concepts automatically generated by Leximancer were suppressed and relevant concepts were created to represent clinical *knowledge* and clinical *practices*. This involved reviewing excerpts that represented relevant concepts and merging those that were conceptually similar. For instance, the concept, ‘clinical knowledge’, was created by merging, ‘antibiotic guidelines’; ‘card’ and ‘placard’, denoting the STI Testing Tool; ‘electronic therapeutic guidelines’ and ‘ETG’; ‘guidelines’; ‘Harrison’s’, as in the principles of internal medicine [80]; ‘Murtagh’s’, as in the reference guide on evidence-based primary care [81]; ‘protocol’; ‘Red book’, which is the Royal Australian College of General Practitioners guidelines for preventive activities in general practice [82]; ‘STI Testing Tool’; ‘TGA’ (or the Therapeutic Goods Administration); and ‘therapeutic guidelines’. Similarly, the concept, ‘clinical practices’, was created by merging, ‘screen’; ‘screening’; ‘test’; ‘tested’; ‘testing’; ‘tests’; ‘treat’; and ‘treatment’. Guided by previous research [78], thirty new user-defined concepts were profiled to examine the concept maps of ‘clinical knowledge’ and ‘clinical practices’ – this number was selected to avoid diluting the focus of the created concepts, ‘clinical knowledge’ and ‘clinical practices’. Despite the demonstrated value of concept profiling, Young and Denize [77] warned that it moves the analysis away from the overall concepts and themes revealed by the material, to one focused on a smaller part of an already relatively small dataset. Although concept profiling helps to focus researcher attention to pertinent concepts and themes, the approach also risks leaving more general concepts and themes unexplored. Given this study focuses on the translation of knowledge into practice, concept profiling was deemed appropriate.

Second, the interview transcripts were tagged. Unlike thematic coding, in which topics or ideas deemed pertinent to particular phenomenon are identified [83, 84], tagging within Leximancer helps to compare the conceptual content of different documents [85]. Guided by the tri-focal theoretical lens, the transcripts were dichotomously tagged to categorise the GPs (see Table 2 for detail on how each theory was operationalised in this study). For instance, institutional theory directs attention to the inherent and relatively resilient aspects of social structures, suggesting the more embedded an individual to their social context, the less likely their capacity to change [38]. As such, assuming that seasoned (rather than neophyte) GPs would be relatively more socialised by institutional logics, GP interview transcripts were categorised (or tagged) by: years in general practice (< 10 years or > 10 years), year of graduation (< 1982 or > 1982), and whether the GP was a Fellow of the RACGP (FRACGP = Yes or No). These tags were used because, reflecting institutional theory, the more experienced GPs who were Fellows of their professional body were assumed to be more institutionalised, relative to their less-experienced counterparts who were not Fellows of this body. Similarly, cognisant that situated change theory views change as emergent over time, the GP interview transcripts were categorised by whether the interview was conducted at stage one of the study, or stage two. This tag was used because situated change theory recognises the importance of time to detect patterns in attitudinal and/or behavioural change. For comparative value, dichotomies were determined by ensuring approximately equal proportions of GPs, where possible.

**Table 2:** Theoretically-Informed Document Tags

Theory	Tag	Rationale	Dichotomies	Participants
Agency theory	GP would increase STI testing if subsidised by the government as a Medicare service	Recompense for clinical practices is likely to further the GP’s self-interest	Probably / Definitely	8
			Probably not / Definitely not / Unsure	13
	GP would increase STI testing if there was an incentive payment to GPs for each STI test performed	Recompense for clinical practices is likely to further the GP’s self-interest	Probably / Definitely	8
			Probably not / Definitely not / Unsure	13
Institutional theory	Years in general practice	The longer a GP has been in general practice, the more embedded they are likely to be to their profession and/or organisation	<10 years	9
			>10 years	12
	Year of graduation	The longer a GP has been in general practice, the more embedded they are likely to be to their profession and/or organisation	<1982	7
			>1982	14
	FRACGP	Affiliation with the professional body is likely to suggest greater embeddedness to the profession and/or organisation	Yes	10
			No	11
Situated change theory	Data collection stage	Transformation is more likely to be identified longitudinally	Stage 1	21
			Stage 2	20

Together, concept profiling and tagging the transcripts helped to view the research material through the tri-focal theoretical lens. This involved two techniques. First, as per previous, thirty concepts were profiled using the themed discovery setting, ‘Concepts in EACH’, to ‘discover concepts that distinguish… categories from one another’ ([86], p. 154). For instance, reflecting agency theory, which ‘reminds us that much of organizational life, whether we like it or not, is based on self-interest’ ([8], p. 64), the setting – ‘Concepts in EACH’ – was used to compare GPs who *would* increase STI testing if subsidised by the government as a Medicare service, with those who would *not* increase STI testing (see Table 2). Similarly, this setting was used to compare GPs who *would* increase STI testing if provided with an incentive payment for each STI test performed, with those who would *not* increase STI testing. Second, concepts close to a tag (e.g., the tags to identify those GPs with less than, or more than ten years in general practice) were examined to determine whether they reflected a shared or an individual experience – this was aided by sourcing excerpts that epitomised the concept under investigation. This helped to identify the concepts that were relevant to the study and as such, should be included within the map. Conversely, concepts deemed irrelevant were suppressed.

Relevant concepts were then analysed in two ways. First, the proximal distances between tags and concepts were considered. Second, excerpts associated with the created concepts, ‘clinical knowledge’ and ‘clinical practices’, were sourced and examined in relation to each tag. Reflecting previous research [79], because the two created concepts were common to all maps, there was considerable similarity between them. Yet, of value here is the ability to unveil similarities and differences between the maps, as well as between the thematic and lexical analyses.

## Results

Following a description of the GPs who contributed to this study, this section presents findings from the theoretically-informed analyses of the interview transcripts. For clarity, the section presents findings that were informed by the use of the agency, institutional, and situated change theories, respectively – furthermore, each of these three subsections commences with a brief overview of the theory and includes signposts to denote: findings associated with the thematic analysis; findings associated with the lexical analysis; as well as a summary of key findings.

### Participants

Twenty-one GPs participated in the study, including four registrars, or GPs-in-training (female: 14; mean age: 45 years; age range: 27–69 years) – however, for reasons unknown, one GP was not available to participate in the second stage of data collection. On average, the 21 GPs had been in practice for over 16 years. Although all but two had graduated in English-speaking countries (the exceptions being Egypt and India, where English is not the most common language), approximately half conducted a portion of their consultations in a language other than English (52.4%). The GPs practiced in diverse settings – while one practiced part-time in a solo-setting, another practiced in a group-setting comprised of 15 fulltime GPs. Although five practiced in an independent practice, on average, there were 4.5 fulltime equivalent GPs at the practices represented in this study. Most of the practices also employed a Practice Nurse (61.9%), and all but two of the practices were accredited. Approximately half of the practices bulk-billed all patients, accepting government benefits as full payment for a service (52.4%). Most of the remaining practices bulk-billed recipients of government-benefits and/or children under 16 years of age. All but four practices were also teaching sites, offering training opportunities chiefly to GP registrars and/or undergraduate students. All GPs used computers as part of their practice, primarily for prescribing, placing orders, and maintaining medical records.

### Agency theory

Agency theory suggests the primary driver of organisational change is the continued process of negotiation between the principal – in this case, the organisations and patients to who the GPs were accountable – and the agent – that is, the GPs. The theory emphasises the role of incentives and self-interest and draws attention to the strong professional identity within medicine.

#### Thematic analysis

A thematic analysis of the research material suggests that sexual healthcare occupied a trivial place, relative to general practice (*sensu lato*). Sexual healthcare was considered a comparatively small part of the GP platter of responsibilities; these consultations were infrequent and this in turn could hinder the recall of evidence-based sexual healthcare. Although this makes a compelling case for resources like the STI Testing Tool, securing GP attention – given competing priorities – might be difficult:

I can pretty much put my hand on… information about lipids, cholesterol, diabetes, and hypertension, and probably asthma any day of the week. But other things, I don’t know where they are, tossed out, or thrown away or something like that, because you’re not dealing with them in that same repetitive way.

Integrating information into general practice appeared to be a challenge. This was partly due to limited time. GPs spoke of having limited opportunity to develop a trusting relationship with patients and provide appropriate care. They had to manage their limited time frugally:

compared to diabetes, cardiovascular, strokes… those hardcore medical things, STIs, even though it’s very prevalent… I don’t want to spend lots… of time on that because I might see… one case a month, one case every fortnight… not… ten cases a week.

Another determining factor was the perceived value of a resource. Some GPs suggested they worked within a paradigm that did not correspond well with resources that compartmentalised healthcare:

nurses are trained to follow protocols and go by guidelines and they don’t sway outside of that, whereas a doctor is… taught to think more laterally… that’s why doctors are not good at following flowcharts and guidelines… they explore the issue and make a decision about what they think should be done, not according to whether this is a yes or no.

This was affirmed by others who spoke of the sensitive nature of sexual healthcare. Sexual health consultations could reveal instances of infidelity and abuse. As such, these consultations were not always determined by clinical guidelines, but rather, by a GP’s personal interest in the area:

there was one girl recently who was very shy about it and so I just backed off and… I made some reason to call her back because I just felt there was something else going on and there was. She’d been abused as a child, which was why she wasn’t comfortable talking about it… we… got some counselling and then [I] called her back to see how she was going a week later and then said… ‘I appreciate this is really tough, but would you like the peace of mind of knowing?’

#### Lexical analysis

**Fig. 2:**
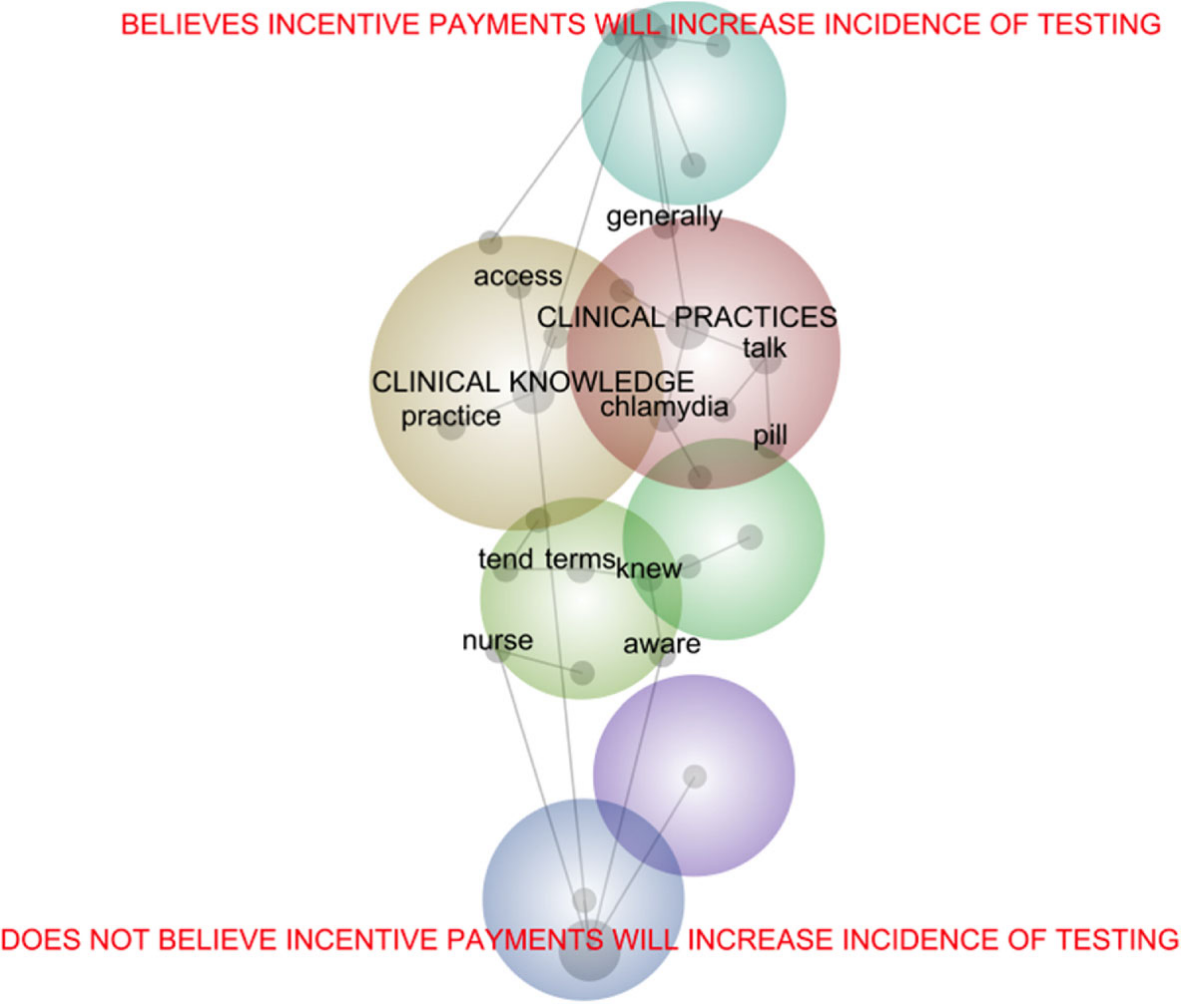
Concept Map of GPs categorised by Interest in an Incentive Payment to Increase STI Testing

**Fig. 3:**
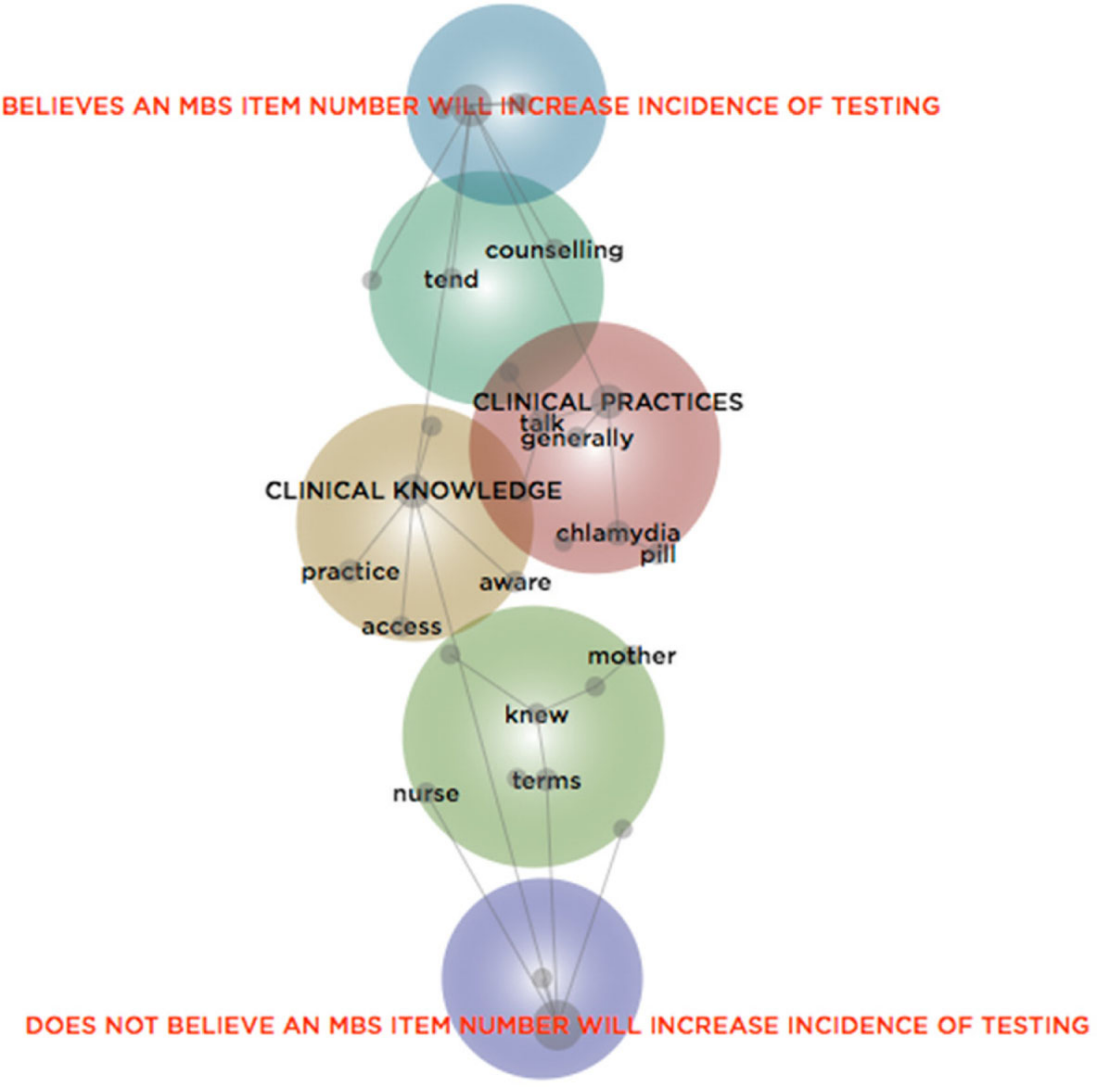
Concept Map of GPs categorised by Interest in an MBS Item to Increase STI Testing

A lexical analysis was conducted by developing two concept maps. Within the first, the 21 GPs were categorised according to whether they would increase STI testing if an incentive payment was offered (see Fig. 2); within the second, the GPs were categorised according to whether or not they would increase STI testing if subsidised by the government (see Fig. 3). Both figures suggest that the concept, ‘clinical practices’, and to a lesser extent, ‘clinical knowledge’, are closer to the category of GPs whose use of sexual health tests would increase in response to an incentive payment and/or a government subsidy. This suggests that those whose clinical practices could be incentivised were more inclined to speak of screening, testing, treating, and/or the resources required to support these practices. For instance, these GPs spoke of the ways they ‘generally’ broached the sensitive topic of sexual health and their delicate use of sexual health ‘talk’ to engage a patient in a sexual health consultation:

You tend to fire warning shots that you’re going to ask a question that might be a personal one, and if they back off at that point, then you can address it differently.

These concept maps and the aforesaid excerpt indicate that GPs who spoke of an incentive were inclined to speak of the complex and processual nature of sexual healthcare, which required time, and relatedly, due recompense in recognition of this time. However, this is not to suggest a uniform preference for incentivised sexual healthcare. Some GPs recognised that this was likely to be associated with protocolled care, which may not bode well with its processual nature:

You’re not doing everything by flowcharts and guidelines. You’re making an assessment about that person as an individual and their risks for things… doctors make decisions all the time that aren’t according to guidelines.

Furthermore, incentive payments for sexual healthcare were associated with discourse around, ‘access’. For instance, some GPs commented on the need for information that was obtainable and user-friendly; while others considered how an increase in sexual health testing might increase consultation times and affect patient throughput:

that’s actually quite extensive. If you really do the HIV [human immunodeficiency virus] pre-test counselling properly, or as it’s done in the books, that alone could be a whole consultation.

#### Key findings

Collectively, the findings from both the thematic and lexical analyses connect with agency theory in three ways. First, identity as a *general* practitioner might reduce the attention awarded to health issues that are considered infrequent. General practice might be dominated by perceived priorities; as such, GPs might require additional guidance on the conditions they have limited exposure to. Second, the findings allude to the influential role of self-interest – for instance, the need to manage time – as well as personal interests – like, the need to be primarily familiar with evidence-based practices deemed to have direct relevance to current patients. Irrespective of the desires of the principal – that is, the organisation in which the GPs worked or their patients – the agents – namely, the GPs – largely self-organised around particular interests. Third, the findings draw attention to the difficulty principals can face when attempting to influence the behaviour of agents who are accustomed to particular practices. The challenge for the principal is to enable those who ‘think [they]… sort of know what to do’ to reappraise well-trodden cognitive paths:

you get set in the ways of patterns… Unless you graduated last year… you’re set in your ways of investigating and treating and it takes a little bit more… to change things.

### Institutional theory

Institutional theory encourages us to consider the, ‘processes used by organizations to adapt to the political, cultural, and social demands of their environment and gain legitimacy in the eyes of stakeholders’ ([87], p. 84). This study considers these processes from the perspective of the GP, thereby foregrounding institutional work, rather than institutional logics [88]. Although these organisational processes can constrain change, they can also help individuals to understand how the organisation functions and identify viable ways to introduce and sustain change.

#### Thematic analysis

A thematic analysis of the research material suggests that, for the most part, the GPs were unable to describe the ways their work-context functioned. Notwithstanding cursory references to patient-booking systems, bulk-billing, and the use of information technology, they seldom detailed the ways their practices were organised, administered, and/or governed:

If you’ve got good software, they’ll actually put those templates in for you, so that when you’re doing investigations or tests… you’ve already got that in place.

Although a few GPs described occasional opportunities to shape organisational practices, there was little active involvement in organisational life:

We have a larger practice meeting with everyone including the receptionists… maybe twice a year… There, we could also sort out issues of how the practice runs… how results are dealt with, how the receptionists communicate with us and vice-versa, and how appointments are run and whether there are things that can be changed.

Notwithstanding the aforesaid excerpt, the GPs seldom recognised an association between their work-context and their professional role. With few exceptions, general practice was typically described as occurring behind closed doors, somewhat dissociated from factors beyond the clinic. For instance, when asked whether organisational factors influenced their capacity to provide evidence-based practice, a GP stated:

The practice doesn’t do much at all – it’s the GP.

According to the GPs, management fundamentally occurred at a micro level as they attended to patients’ clinical needs. They spoke of filtering the information that patients presented; eliciting further information when required (or at least, attempting to); negotiating patient relationships; diagnosing and treating conditions; and helping patients to manage these conditions:

The actual style of management will all vary… Some colleagues of mine are fantastic; they’ll spend half-an-hour just really counselling the patient, whereas some colleagues might say, ‘Well this is the result, this is the treatment, see you later’… or, ‘I can refer you to a further clinic’.

Given the GPs’ key role as clinicians (even among the five sole clinicians who managed their own practices), the paucity of detailed references to institutional life might be attributed to a range of reasons. For instance, the GPs might have been unfamiliar with, overwhelmed by, and/or uninterested in the organisation in which they worked, which was obliged to adhere to national standards. Similarly, they might not have considered the operation of the practice within their role, preferring to distance themselves from managerial responsibilities:

Every year I say this is the last year I’ll go through accreditation.

Although it is not the purpose of this article to hypothesise reasons, these findings do not portray the GPs as inextricably connected to their practices. But rather, they seem somewhat disconnected from institutional work, particularly those practices that maintain the heavily-regulated institution of medicine.

An exception however pertains to one GP who used her institutional savoir faire to promote sexual health. She was the only female GP in a practice of male GPs who were largely uncomfortable with sexual healthcare. Her expressed interest in sexual healthcare offered some relief to her colleagues who were content (if not relieved) to refer most, if not all sexual health cases to her. In addition to allowing her opportunity to develop her expertise in this area, it also helped to ensure that evidence-based sexual healthcare was delivered by the practice. This suggests the benefit of institutional savviness, whereby familiarity with the practice and its modus operandi can be used to introduce and sustain health services that might not be offered otherwise:

The blokes have all been very supportive because they’ve said, ‘Fantastic, you can do all that’… they’ve put an advertising campaign out so that people are aware that I’ve got sexual health and reproductive health interests.

#### Lexical analysis

**Fig. 4:**
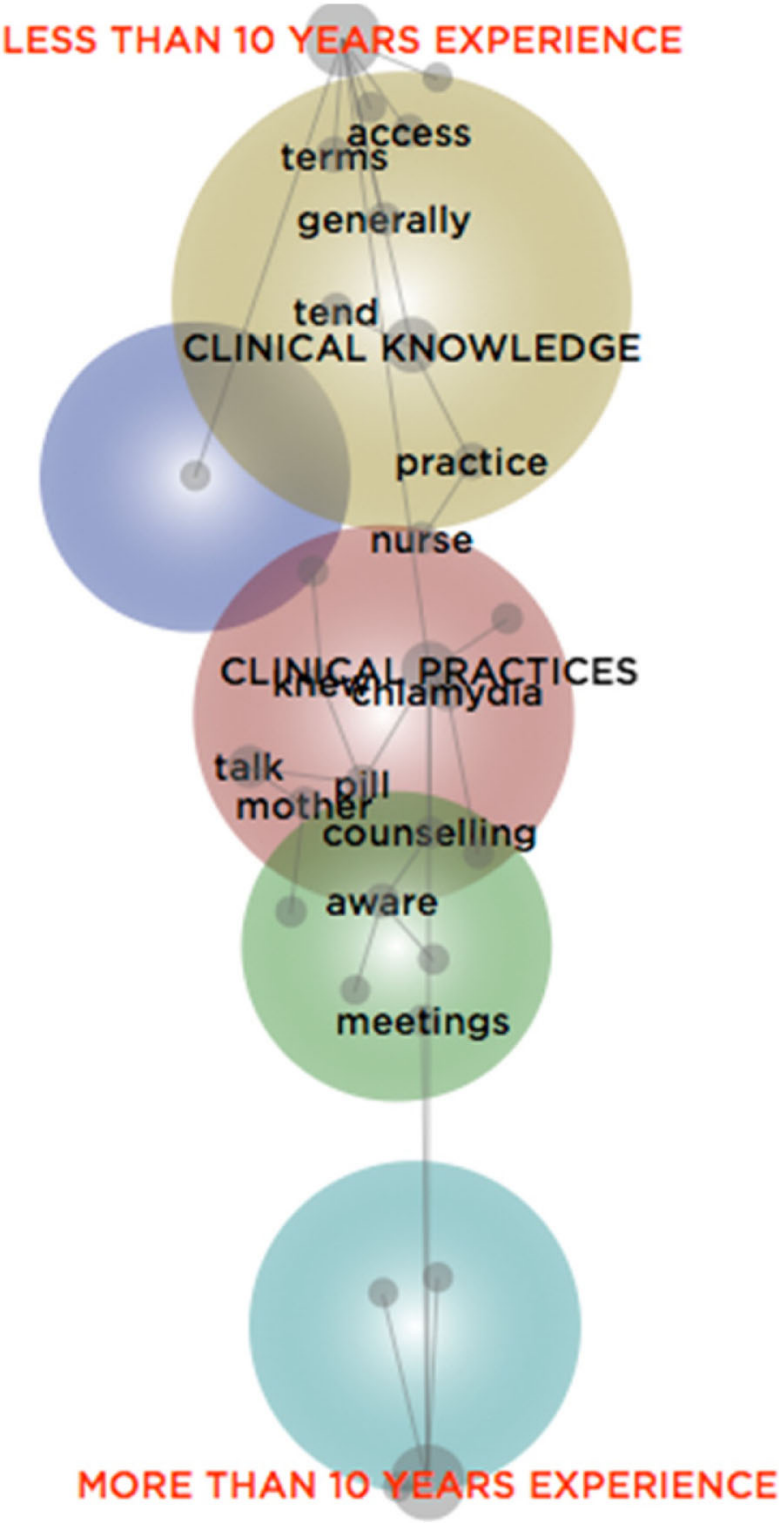
Concept Map of GPs categorised by Years of Experience

**Fig. 5:**
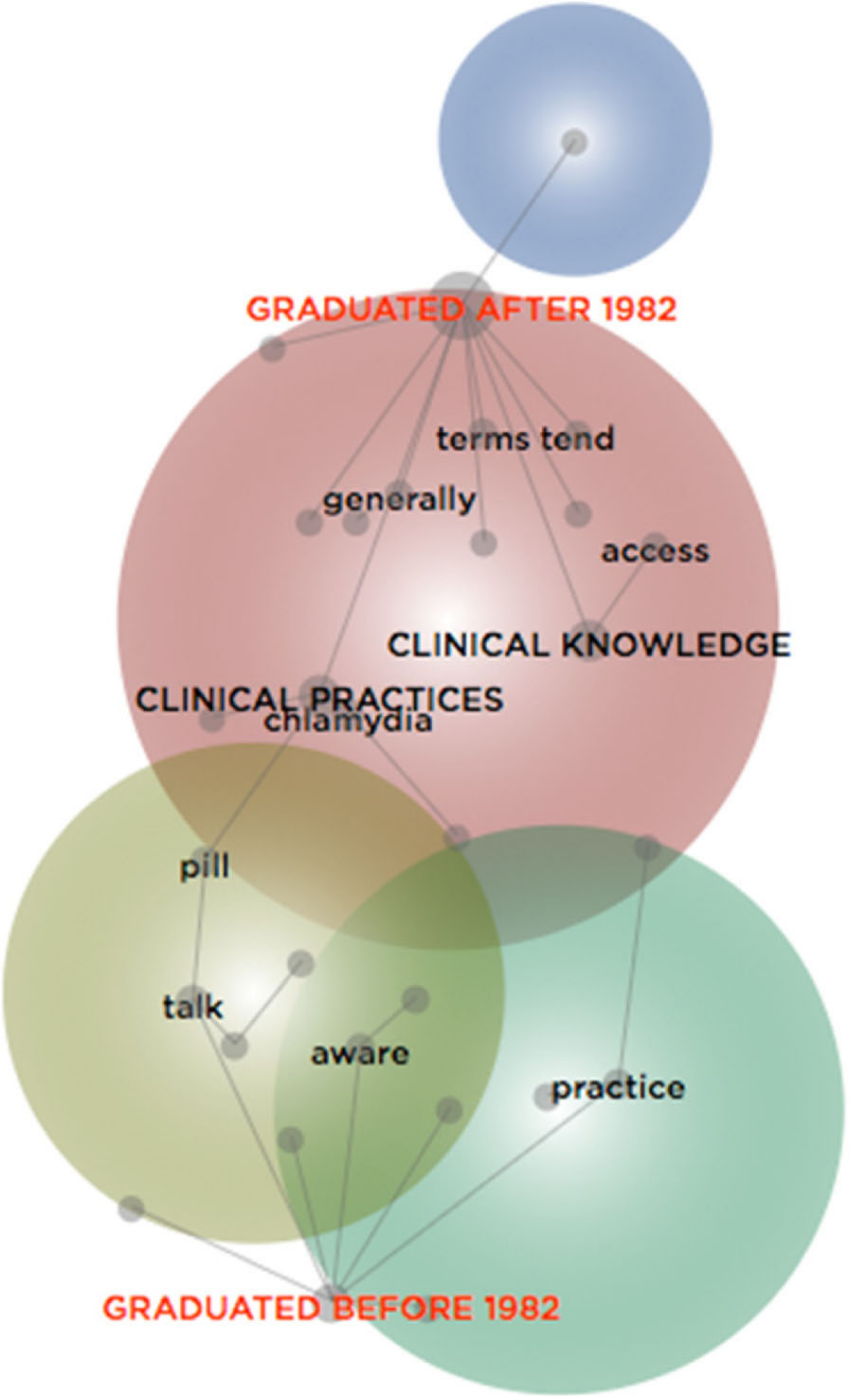
Concept Map of GPs categorised by Year of Graduation

**Fig. 6:**
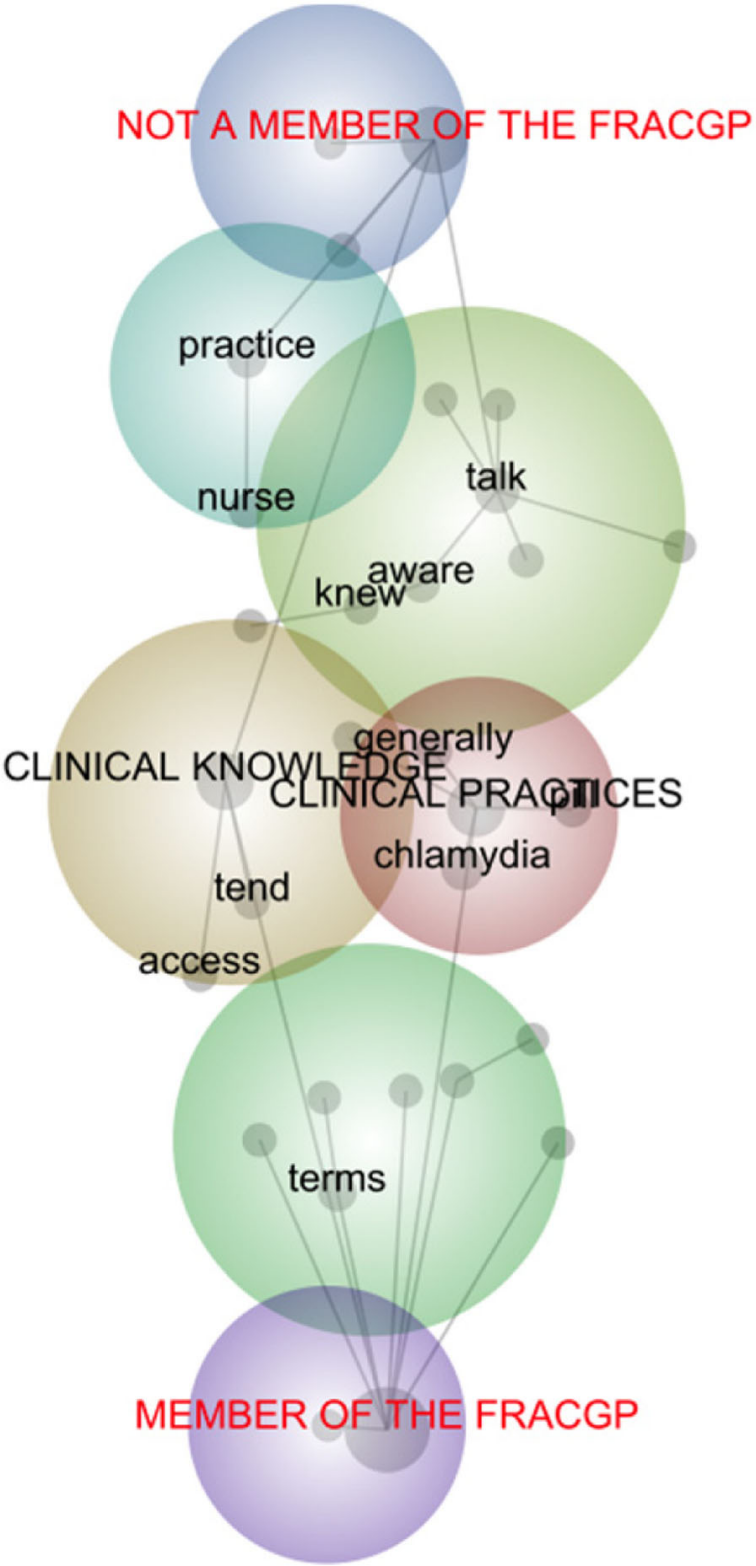
Concept Map of GPs categorised by FRACGP

Complementing this thematic analysis, a lexical analysis was conducted by developing three concept maps. Within the first, the 21 GPs were categorised according to their years of experience (see Fig. 4); within the second, they were categorised according to their year of graduation (see Fig. 5); and within the third, the GPs were categorised according to whether or not they were a Fellow of the RACGP (see Fig. 6). Each map is interpreted in turn.

Within Fig. 4, the concept, ‘clinical knowledge’, is closer to the category of GPs with less than ten years of experience, while the concept, ‘clinical practices’ is closer to the category of those with more than ten years of experience, and thus, more likely to have established practice or routines. This suggests those with less clinical experience primarily referred to clinical guidelines, resources, and other repositories of knowledge, while those with greater experience spoke of screening, testing, and treating. Furthermore, these experienced GPs often noted the value of ‘meetings’ in which they would confer with, and learn from clinical peers, rather than the aforesaid knowledge repositories:

I go to [Division] meetings and sometimes the… issues [discussed]… are probably very compact. I think that’s probably been the most useful.

we could do a lot better at having regular clinical meetings… we’ve got about six doctors [here]… and there isn’t a day goes by when we’re all here at once.

Figure 4 also situates the concept, ‘practice’, closer to the category of GPs with less than ten years of experience. This suggests that those with relatively less time within the profession spoke of ways in which they performed and enacted their role. Sometimes, their ‘practice’ abraded against organisational requirements, thus revealing connections between the individual clinician and the institution they represented:

[The] quality cycle involves a clinical audit… It involves an audit at the beginning, and then an audit to see if the changes everybody agreed on have… been implemented… We do it, but we could do it better…We haven’t incorporated it that well enough into the culture of the practice, so a few people do that, but it’s not generalised.

Figure 5 locates the concept, ‘tend’, close to the category of GPs that graduated after 1982. These younger GPs largely referred to the ways they readily sourced information, independently, to guide their practice. These individual efforts reveal a disconnect from the practice they were affiliated with, and the clinical colleagues therein. Rather than refer to organisational resources, including the colleagues therein, they were more inclined to use familiar resources beyond the organisation, including teaching aides and mentors:

my supervisor’s got a lot of information… I can just call him … he’s my main resource… he’s got quite an evidence-based mind, so I feel comfortable asking him what he thinks about something because often it’s not just his opinion; he would have read a lot and done a lot of research on the particular topic… if you notice over… time that [a doctor]… always prescribe[s] an antibiotic for upper respiratory tract infections, or they always give a steroid for itchy skin… you kind of go, ‘Well, obviously you’re just doing that because you don’t want to think about it’. So, I’ve just stopped asking those kinds of people.

The aforesaid excerpt helps to clarify this penchant for familiar resources that lie beyond the immediate organisational domain – not only why particular resources were used, but how their value came to be recognised. As the excerpt suggests, some GPs thoughtfully observed whether their colleagues dutifully complied with what their profession or their organisation expected, and how they deviated from these expectations to deliver what they understood to be evidence-based care.

Figure 5 places ‘clinical practices’ and ‘clinical knowledge’ thematically closer to the category of GPs who graduated after 1982, as these concepts are located within the largest theme. This suggests these relatively inexperienced GPs made greater reference to varied clinical resources, discerning appraisal processes to determine their value, the use of preferred sources of knowledge, and the delivery of sexual healthcare:

I don’t use guidelines as a backup tool; I use research as a backup tool… guidelines come from somewhere, and unless you know exactly where they come from – it’s a summary… When someone asks you for proof, I don’t want to use a tool as proof.

Conversely, those who graduated before 1982 are connected with discourse on being ‘aware’. Cognisant of the potential fragility of the patient relationship, they were conversant with how to broach, discuss, and deliver sexual healthcare. Embedded within their profession, some were unable to precisely articulate how they developed these skills – yet they recognised the ways in which context, including the patients they consulted, shaped what they did and how they did it:

I don’t pretend… that I’m an expert… but I certainly am aware of, especially in young people, the potential problems.

here, it’s pretty much suburbia; it’s not like in Kings Cross [a city suburb]… we are aware of the STDs [sexually transmitted diseases] among certain age groups… So we just raise the index of suspicion and screen them if it’s possible.

Together, Figs. 4 and 5 suggest a form of cognitive inertia is associated with discourse on sexual healthcare, when considering the GPs according to their years of experience and year of graduation. Those with over ten years of experiences and (related to this) those who graduated before 1982 seemed to own the language on ‘clinical practices’, relative to their inexperienced counterparts. This might indicate a greater preference for institutional ‘rules of thumb’ ([22], p. 489) than different forms of ‘clinical knowledge’. What these findings reveal is that experience is a powerful predictor in terms of engagement with clinical guidelines and resources. Although inexperienced GPs might frequently draw on external resources to supplement their knowledge-base, this tendency perhaps for some, fades over time.

Within Fig. 6, although the concepts, ‘clinical knowledge’ and ‘clinical practices’, are equidistant between the categories of GPs who are, and are not Fellows of the RACGP, it is only the former group that has a direct relationship to discourse on both concepts. This suggests that Fellows of the RACGP made greater direct references to both clinical resources and their use. This is supported by the position of, ‘access’, which is closer to the category of RACGP Fellows:

it’s very important that we have access to clear and up-to-date information on things like chlamydia, gonorrhoea, hepatitis, because it’s all so daunting for the patient and they’ve just got a horrible sinking feeling.

to have things online can be very helpful in that sense, because at least you can do ongoing education.

#### Key findings

The thematic and lexical analyses reveal the relevance of institutional theory by highlighting the ways in which embeddedness within an organisation and a profession can shape clinical practices. Although the thematic analysis largely suggests the GPs were somewhat disconnected from institutional life, it also revealed the value of institutional savviness. Extending this finding, the lexical analysis illustrated how experience can be both an asset and a limitation when attempting to engage with, and keep abreast of evidence-based practices.

### Situated change theory

The situated change theory suggests that change is emergent and evolves over time. Furthermore, subtle change is no less significant than large-scale, deliberate, orchestrated change [9].

#### Thematic analysis

Following the thematic analysis, this was demonstrated by several GPs, particularly those who had a personal interest in sexual healthcare. While some spoke of adapting clinical guidelines to their clientele, others participated in self-initiated study groups to review and debate sexual healthcare resources:

me and a couple other of my colleagues from a different practice… are in a study group and we look up guidelines for each sexual health problem… and then we’d go through that and then I’d base my practice largely on that.

Similarly, a young female GP spoke of availing herself to information from an array of sources. In addition to clinical guidelines and professional development workshops, she consulted ‘as many people as possible’. Furthermore, she reflected on these discussions and identified feasible strategies to improve her own practices. For instance, inspired by a ‘long and… open’ discussion with colleagues, she recognised some of the awkwardness she experienced when consulting mature-age male patients about sexual health; this was the impetus for personal change. She endeavoured to better understand her practices, harness opportunities to deliver sexual healthcare to this cohort, and gauge improvement:

I used to photograph myself after each consultation to see how red I’d gone to… sort of mark how red I was, because I just felt very uncomfortable… now I don’t blush at all… I’m still not fully, completely comfortable, but I’m much better than I was.

Although multiple sources of information helped this GP to transform her practice, others spoke of information-overload. Some grew indifferent towards the resources they received from the government, not-for-profit, or corporate sectors; this was because the resources needed time and concentration – assets that were largely limited. Such indifference might suggest a retreat to well-trodden cognitive paths:

I did have a look at [the STI Testing Tool]… and probably I was a little bit more aware for the first little while, but I think I’ve slipped back into my old habits, to be honest.

This reliance on familiar practices is supported by GP responses to the clinical vignettes they were presented with on sexual healthcare. Although most appropriately indicated they would screen the patient for chlamydia (90%) and the Hepatitis B virus (65%), many also inappropriately noted they would screen for gonorrhoea (65%) and HIV (70%). The suggested use of these unnecessary tests was largely consistent across both stages of data collection. An interpretation here is that change does not occur instantly, but rather is characterised by a series of forwards and backwards mini-steps that require time to fully emerge, if at all.

A thematic analysis of the research material suggests that limited responsiveness might be curbed using novel channels to communicate information, like public health campaigns and sexual health training. Some GPs indicated greater interest in sexual healthcare due to broad initiatives to promote sexual health:

It’s just become a lot more in journalism and the pathology department newsletters.

These initiatives helped to ensure that sexual healthcare remained on the GP-radar. Similarly, sexual health training enabled the GPs to delve deeper into relatively unfamiliar territory. They had opportunity to practice skills that might have remained out-of-form:

we had a… series of clinics that you could self-place in sexual health clinics, just sitting in and sometimes they’d let us consult… sitting in with the sexual health registrars and… specialists and… learning from them.

#### Lexical analysis

**Fig. 7:**
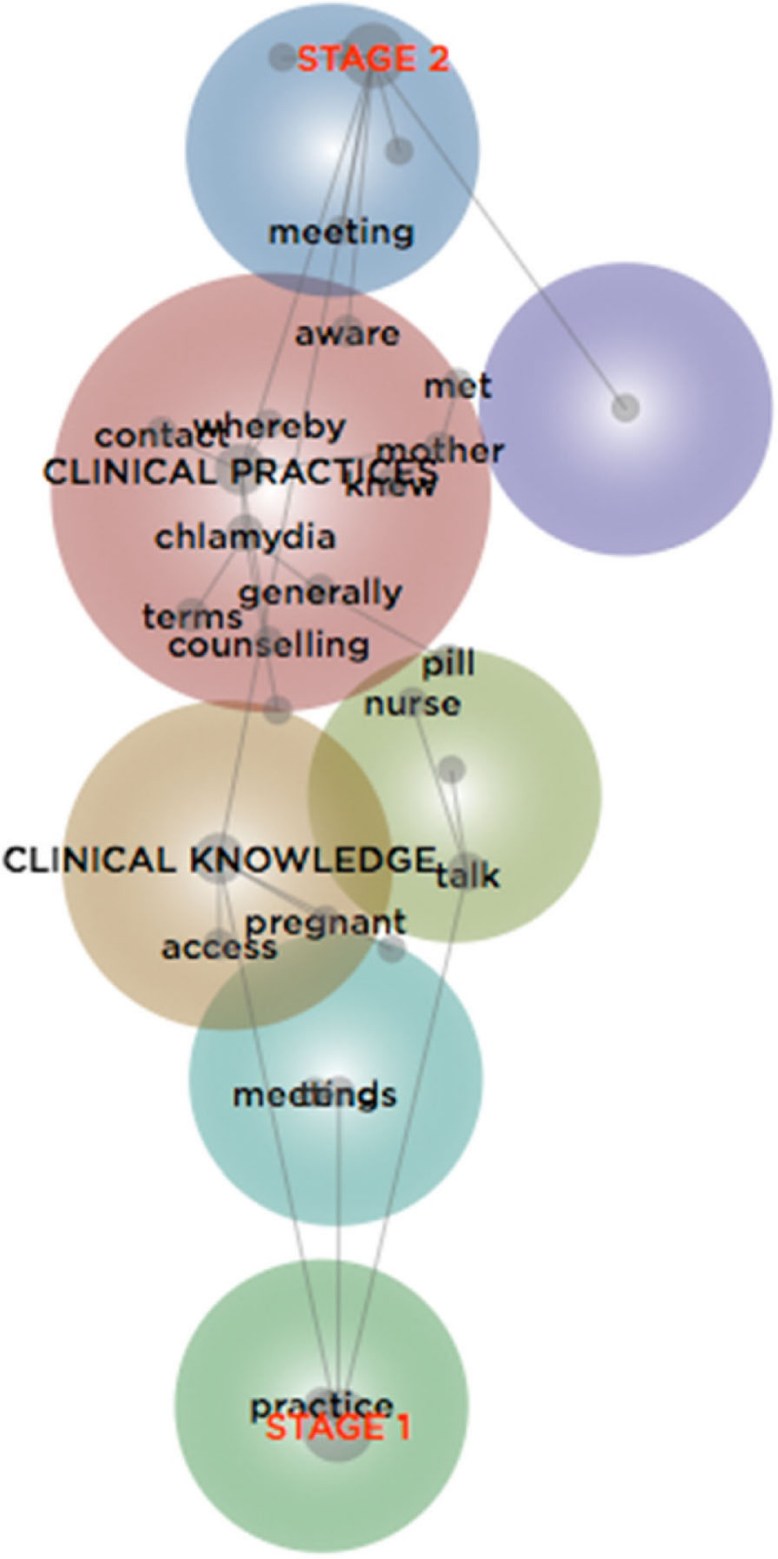
Concept Map categorised by Data Collection Stage

To examine change over time, a lexical analysis was conducted by developing a concept map, in which the research material was categorised according to the stage of data collection (see Fig. 7). The map locates the concept, ‘clinical knowledge’, closer to the category denoting the first stage of data collection, while, ‘clinical practices’, is closer to the category denoting the second. This suggests the GPs’ utterances had a greater focus on artefacts of knowledge, like guidelines, during the initial interview, at which they were presented with the STI Testing Tool, while there was greater focus on the delivery of healthcare, during the subsequent interview. Curiously, the concept, ‘practice’, is located at the tag associated with the first stage – it is worth noting that this concept, unlike ‘clinical practices’, was not created by merging conceptually similar concepts; but rather, it represents unaltered ‘collections of words that generally travel together throughout the text’ ([48], p. 9). An examination of this concept indicates that, during the initial interview, the GPs largely described intuitive, routinised practices, whereby the delivery of sexual healthcare followed well-worn cognitive paths:

you’ve seen this before and you know how to deal with it, so… unless… the treatment’s not working, then… I think it’s easy… the more time you spend in general practice.

Some GPs indicated that over time, they awarded greater primacy to their internal repositories of knowledge, except when uncertainty prompted a search for external knowledge. Knowledge thus became embodied in practice:

you read about the [guidelines]… and then they just become part of my embedded practice

I certainly use the sexual health clinics… just ringing them and asking them for advice.

The concept, ‘Clinical practices’, which denotes screening, testing, and treating, is relatively more associated with the second round of interviews, which occurred three to four months after the GPs were provided with the STI Testing Tool. This is not to suggest their practices changed; but rather, that their language changed, whereby there was greater focus on how they performed their role and enacted their knowledge:

I had a patient who was a man who had sex with men. I was at a loss to know what to test for – here it is [picks up the STI Testing Tool]… I remember thinking, ‘Oh my God, where am I going to find out what’s the appropriate test?’

They described how the initial interview, and the anticipation of the second, prompted different observations of the self and different conversations with others. This is reaffirmed by the concept, ‘meeting’, which refers to meeting with the researcher, rather than ‘meetings’ with clinical colleagues:

immediately after our meeting last time, it was more in the forefront in terms of actually approaching patients and actually making it part of the consultation, if that’s not what they came in for, and… I thought [about whether] it was appropriate to at least bring up the topic, even if we didn’t do anything at that consultation.

I put the effort more in taking more sexual history, just asking people about the relationships and their partners and if they’ve changed partners or had more than one partner. I certainly aim to do it more, I’m aware of it more.

Yet the improvisations that were triggered, in part, by the novelty of the STI Testing Tool and interviews with the researcher, sometimes paled against other contributors to their knowledge, including familiar resources that could be readily sourced within the organisation they worked, and trusted teaching aides. Consider for instance, the concept, ‘aware’, which is positioned close to the category denoting stage two of the study. Within this concept are excerpts in which the GPs spoke of myriad resources they were cognisant of, including ‘Therapeutic Guidelines… [and the] Red Book guidelines’, among others:

I’m still aware of the same resources.

it’s what we have been taught more than anything else. There isn’t a lot in the way of resources that I’ve been drawn attention to.

Reflecting situated change theory, this concept map illustrates the dynamic, gradual, vacillating, and faltering nature of change. The mere exposure to, and comprehension of knowledge artefacts is unlikely to simply align individual behaviours with evidence, as defined by accredited organisations. But rather, knowledge artefacts might help to ignite (even if temporarily): reflectivity and reflexivity [89] around whether and how change might happen, as well as marginal improvisations, with their associated slippages. This reveals the situatedness of knowledge translation – a process of mini defeats and victories, shaped by contextual nuances. The GPs, as registered professionals who largely worked behind closed doors, performed knowledge work within a context that simultaneously structured and freed their thinking and their practices to determine, ‘what things get done and how they are done’ ([90], p. x). This in turn can open additional opportunities for change – as Weick indicated:

The advantages of emergent change include its capability to increase readiness for and receptiveness to… change and to institutionalize whatever sticks… sensitivity to local contingencies… experimentation, learning, and sensemaking; comprehensibility and manageability; likelihood of satisfying needs for autonomy, control, and expression; proneness to swift implementation; resistance to unraveling; ability to exploit existing tacit knowledge; and tightened and shortened feedback loops from results to action ([91], p. 227).

As such, seemingly trivial change in patient consultations, the consideration of resources, or recognition of awkwardness around sexual healthcare might prime the GP towards evidence-based care – not necessarily as defined by accredited organisations, but rather, as a practice that reflects the situated blend of clinician expertise; patient (and potentially carer) preferences; available resources; and the context of care. This is supported by the relative infrequency of those exceptional strategies used by a small number of GPs to explicitly change their sexual healthcare practices. The challenges that GPs face in familiarising with, synthesising, and adhering to ‘evidence’, while managing their workload and accommodating patient expectations, might suggest that these exceptional strategies are precisely that; the exception. Most of their counterparts appeared to inhabit a space that either: primed them to varying degrees of (less exceptional) change; or pushed them back towards the familiar.

#### Key findings

Although the thematic analysis elucidated the factors that influenced change among the GPs, the lexical analysis clarified the dynamic nature of change. This confirms the complementary value of the two approaches.

## Discussion

Following calls a broader approach to knowledge translation research – one that draws from organisation studies [21], this article extends current understandings of knowledge translation in two key ways. First, it applied a tri-focal theoretical lens comprised of agency [8], institutional [92], and situated change theories [9]. Second, it involved the dual use of thematic and lexical analysis. These contributions to extant literature were achieved in a study with 21 GPs to understand the factors that help and hinder the delivery of evidence-based sexual healthcare.

Theoretically, the findings suggest that agency, institutional, and situated change theories each help to reveal different aspects of knowledge translation. Agency theory drew attention to the interests that influenced GP capacity to deliver evidence-based care. Despite their role as *general* practitioners, sexual healthcare did not always remain on the GP-radar, but was often overshadowed by other priorities – including the need to manage their limited time (which was eased by primarily attending to patient priorities), as well as the need to maintain a broad approach to primary care. Institutional theory revealed how familiarity with norms and practices can be advantageous to clinicians. Although not all GPs were familiar with the ways their organisation functioned, some suggested that fluency in organisational processes, including how they and their colleagues made sense of the organisation, and of organising, can be used to ensure that sexual healthcare is delivered by a practice predominantly comprised of GPs who are less comfortable with this area. Finally, situated change theory revealed the importance of incrementalism. For some GPs, gradually changing what they did and how they did it helped to transform clinical practices. Although exposure to multiple sources of information can aid this process, it can also be debilitating. Overwhelmed by the wealth of healthcare information received from the government, not-for-profit, academic, and corporate sectors, some GPs appeared to disengage from resources that were aimed to improve their clinical practices.

Extending this analysis further, agency theory [8] with its focus on principal-agent relationships seems to be at the core of this tri-focal lens, and (like layers of an onion) institutional [92] and situated change theories [9] offer additional layers. Consider for instance, the role of self-interests – although this role is explicit in agency theory, self-interests are also a key driver of change when understood using institutional or situated change theories. The findings from this study reveal how one GP furthered her personal interest in sexual healthcare by welcoming referrals from colleagues who were less comfortable with sexual health consultations. Similarly, the findings demonstrate how personal interests can foster or hinder gradual change. One GP extended his knowledge and skill-base through regular participation in a study group, with the aim to improve his clinical practices; another used photography to obtain biofeedback. Conversely, the findings also suggest that GPs who were overwhelmed by the wealth of clinical resources might fortify their efforts to manage their limited time and disregard the material.

Methodologically, the findings from this study demonstrate the value of both thematic and lexical analyses. Thematic analysis, which was relatively researcher-driver, helped to reveal the relevance of agency [8], institutional [92], and situated change theories [9]. The researchers applied these lenses to the research material to understand their bearing. Lexical analysis, which was relatively machine-driven, served to verify these findings. Relatively devoid of the researchers’ values and assumptions, it helped to critique the inferences drawn from the thematic analysis and avert ill-informed conclusions – this is particularly because the analyses focus on what participants said, rather than a researcher’s (hopeful) interpretation.

For instance, manually coding the research material helped to reveal the influence of time on clinical practices. Time shaped patient consultations, professional development, and the delivery of evidence-based care. These findings complemented the lexical analyses, which did not speak to the significance of time in the same way. Similarly, the lexical analyses help to focus attention on particular GP characteristics, like years of experience. This was achieved by seeding concepts of theoretical importance – namely, ‘clinical knowledge’ and ‘clinical practice’ – and tagging the research material to isolate areas of conceptual significance. This approach helped to understand how the discourse was associated with ‘clinical knowledge’ and ‘clinical practice’ and draw attention to concepts that had particular analytical value, all while reducing researcher-bias. The value of both manual coding and lexical mapping reflects previous research [93].

Despite the value of the findings presented in this article, two limitations deserve mention. First, because GPs were self-selected, there is no claim they constitute a representative sample of primary care clinicians. Second, the reliance on interview transcripts limits the lifespan of the identified findings, particularly because of the potential for social desirability bias.

## Conclusions

Notwithstanding the aforesaid limitations, this study is important for theoretical, methodological, and practical reasons. Theoretically, this study is the first to examine knowledge translation using a framework premised on agency, institutional, and situated change theories. It revealed that the use of clinical information and the delivery of evidence-based sexual healthcare are shaped by the agent (i.e., the GP) and the institution (i.e., their profession and the organisation they are affiliated with); furthermore, the influence of the agent and the institution can be subtle. This finding helps to strengthen an area of research that is largely atheoretical. Related to this, lessons garnered from this study have potential relevance further afield. Methodologically, this study highlights the complementary value of researcher-driven and researcher-guided analysis of qualitative research material. Each revealed different understandings of knowledge translation processes. Practically, this study unveils opportunities to facilitate knowledge translation. More specifically, it suggests that one-off, stand-alone initiatives to promote evidence-based care are likely to have limited impact. Rather, efforts to shape clinician practices should accommodate the potential interrelated influence of the agent and the institution – furthermore, they should recognise that change can be ever so subtle. These findings are particularly timely given the pressing need for healthcare that is both effective and efficient.

## Additional file

Additional file 1 Interview Schedules. Semi-structured interview schedules to collect data at stages one and two. (DOCX 33 kb)
