# Supplementary material for: What can organisational theory offer knowledge translation in healthcare? A thematic and lexical analysis
Source: BMC Health Serv Res. 2018 May 10;18:351. doi: 10.1186/s12913-018-3121-y (PMC5946475; doi:10.1186/s12913-018-3121-y)
Supplement: Supplementary file 1 — Interview Schedules. Semi-structured interview schedules to collect data at stages one and two. (DOCX 33 kb) [file 12913_2018_3121_MOESM1_ESM.docx]

# Additional file 1: Interview Schedules

## Semi-Structured Interview Schedule to Collect Data at Stage One

1. Reasons that patients typically access primary care from this service and the place of sexual healthcare among these reasons
2. Factors that help or hinder the use of evidence-based practice, particularly that pertaining to sexual healthcare – prompts might include:
   1. Awareness of available guidelines (e.g., Redbook, STI Testing Tool)
   2. Use of available guidelines
   3. Use of clinical software tools
   4. Regularity of consulting patients of a particular demographic, perceived or otherwise (e.g., young people)
   5. Degree of difficulty in examining sexual health
   6. Barriers that hinder the delivery of sexual healthcare – prompts might include:
      1. Organisational factors
      2. Patient-related factors
      3. Personal factors
3. Current practices when working with patients to document their sexual history and manage their sexual health (including contraception, immunisation, and the testing of sexually transmissible infections) – invite examples for demonstrative purposes
4. Satisfaction with current practices – prompts might include:
   1. How comfortable are you with your current approach when consulting patients about sexual health?
   2. What would you change about your current approach when consulting patients about sexual health?
   3. What are the strengths of your current approach; how could it be improved?

## Semi-Structured Interview Schedule to Collect Data at Stage Two

1. Perceived change in current practices when working with patients to document their sexual history and manage their sexual health (including contraception, immunisation, and the testing of sexually transmissible infections – be it the type of test(s) or the frequency of testing) – invite examples for demonstrative purposes
2. Practices that have not changed and the associated reasons for this – invite examples for demonstrative purposes
3. Perceived association between current practices and the STI Testing Tool – invite examples to demonstrate the use of the STI Testing Tool
4. Satisfaction with current practices – prompts might include:
   1. How comfortable are you with your current approach when consulting patients about sexual health?
   2. What would you change about your current approach when consulting patients about sexual health?
   3. What factors influence your current practice? Prompt might include:
      1. Time
      2. Comfortability
      3. Access to information
      4. Clientele
      5. Management of the practice
5. Improvements to the STI Testing Tool – prompts might include:
   1. Wording
   2. Format
   3. Location within the clinic
